# Collaborative development and implementation of a knowledge brokering program to promote research use in Burkina Faso, West Africa

**DOI:** 10.3402/gha.v8.26004

**Published:** 2015-01-27

**Authors:** Christian Dagenais, Télesphore D. Somé, Michèle Boileau-Falardeau, Esther McSween-Cadieux, Valéry Ridde

**Affiliations:** 1 Department of Psychology, University of Montreal, Montreal, Quebec, Canada; 2 Knowledge Broker, Société d’Études et de Recherche en Santé Publique, Ouagadougou, Burkina Faso; 3 University of Montreal School of Public Health, University of Montreal Hospital Research Centre (CRCHUM), Quebec, Canada

**Keywords:** knowledge brokering, knowledge transfer, global health, evidence-based practices, evidence-based decision making, Burkina Faso, Africa

## Abstract

Despite efforts expended over recent decades, there is a persistent gap between the production of scientific evidence and its use. This is mainly due to the difficulty of bringing such knowledge to health workers and decision-makers so that it can inform practices and decisions on a timely basis. One strategy for transferring knowledge to potential users, that is, gaining increasing legitimacy, is knowledge brokering (KB), effectiveness of which in certain conditions has been demonstrated through empirical research. However, little is known about how to implement such a strategy, especially in the African context. The KB program presented here is aimed specifically at narrowing the gap by making scientific knowledge available to users with the potential to improve health-related practices and decision making in Burkina Faso. The program involves Canadian and African researchers, a knowledge broker, health practitioners, and policy-makers. This article presents the collaborative development of the KB strategy and the evaluation of its implementation at year 1. The KB strategy was developed in stages, beginning with a scoping study to ensure the most recent studies were considered. Two one-day workshops were then conducted to explore the problem of low research use and to adapt the strategy to the Burkinabè context. Based on these workshops, the KB program was developed and brokers were recruited and trained. Evaluation of the program's implementation after the first year showed that: 1) the preparatory activities were greatly appreciated by participants, and most considered the content useful for their work; 2) the broker had carried out his role in accordance with the logic model; and 3) this role was seen as important by the participants targeted by the activities and outputs. Participants made suggestions for program improvements in subsequent years, stressing particularly the need to involve decision-makers at the central level.

Calls are increasingly being issued for more use of research results by practitioners and decision-makers (1). Despite the many efforts expended over recent decades, there remains a gap between the production of scientific evidence and its use (2–5) . The knowledge brokering (KB) program presented in this article is aimed specifically at narrowing this gap by promoting the use of scientific knowledge to improve health practices and decision making in Burkina Faso. This article presents the different stages of developing the KB strategy and the results of the evaluation of the program's first year of implementation.

The KB activities are part of a larger research program evaluating community-based interventions as well as practices that foster health equity (http://www.equitesante.org/; http://www.equiperenard.ca/). This research program is being conducted in one of the poorest countries in Africa, a continent that continues to have enormous health needs. For example, despite an encouraging reduction over recent years, the maternal mortality rate in Africa is 480 per 100,000 live births, as opposed to 14 per 100,000 in high-income countries; the discrepancy is about the same for mortality rates in children under 5 years of age (107/1,000 vs. 6/1,000, respectively) (6). These situations always disadvantage the most vulnerable (the poor and those living in rural areas), with health gradients seen in all the indicators. The consequences are profound. Estimates suggest that in Burkina Faso around 100,000 children under 5 die every year – about 25,000 of those from malaria – and 2,000 women die of pregnancy-related causes (6).

The primary objective of the first phase of this research program, which involves a participatory planning process, was to evaluate community-based interventions carried out in the Kaya district in order to compile evidence on their effectiveness in promoting health equity. We define community-based interventions here as all interventions (policies, projects, and actions) that target populations directly, as close as possible to where they live, and that are implemented with members of the community, whether formally organized or not. This first phase is intended to provide input into the development of a second phase at a broader national level.

In January 2012, practitioners, decision-makers, and the research team met to describe the theory underpinning the interventions to be evaluated (mutual health organizations, or MHOs,^1^ free healthcare, maternal health services, etc.). Four groups were created around different themes – maternal health, malaria prevention, free healthcare, and family planning – to formulate the research questions. In plenary discussion sessions, participants learned about the processes each group had gone through to describe the intervention model that applied to their research domain, and all were able to raise evaluation questions. Those evaluation questions were brought forward in discussions among researchers about the relevance and the potential for responding to the evaluation questions raised by actors in the field. We noted that some of these questions were already being addressed in studies being conducted by researchers in the team, and that others had already been answered in the literature. These latter questions guided the knowledge broker's work. For example, the question raised by the malaria prevention group – ‘What consciousness raising methods have positive impacts on public awareness?’ – was the first one the knowledge broker addressed using documentary research.

All members of the research team are involved in the KB program: six Canadian researchers from three universities (Montreal, McGill, and Ottawa); one French researcher from the Institut de recherche pour le développement (Institute of Research for Development); and four Burkinabè researchers from the University of Ouagadougou, the Institut de Recherche en Sciences de la Santé (Health Sciences Research Institute), and the Société d’Études et de Recherche en Santé Publique (Society for Public Health Studies and Research). Aside from the researchers, the program includes a knowledge broker; health professionals (e.g. the district medical director and nurses); community-based organizations (e.g. international and local NGOs, and MHOs); and local, regional, and national policy-makers (e.g. mayor's office, regional departments of health and social action, and directors of national public health programs).

The gap between the production of scientific evidence and its use is due mainly to the difficulty of bringing research results to health workers and decision-makers at the local, regional, and national levels, where it could inform practices and decisions in a timely manner. Just because relevant information exists does not guarantee it will be used by health system decision-makers. They also need to see it as helpful in addressing a problem they are experiencing, and there need to be appropriate means by which they can access it (5, 7, 8). The simple dissemination of knowledge alone has relatively little impact on its uptake (9). Results are often presented in a technical vocabulary not geared toward the lay person and require considerable time for someone not trained in basic research to read and understand (10). Consequently, many authors advocate tailoring the format in which results are disseminated and accompanying the presentation of research results with clear paths for action and decision making (11–17) . Although researchers have a role to play in presenting their results to potential users, not all researchers have the tools or expertise required to transfer knowledge to user environments (18–20) . Acquiring such competencies and developing the resources needed to transform their research results into usable guidance for decision-makers are major challenges for researchers.

Employing knowledge brokers to facilitate the use of research results is increasingly recommended (21–25) . This strategy is intended to support evidence-based decision making in the organization, management, and provision of health services (26). In an exploratory review of the current state of knowledge on this new strategy, we identified 19 empirical studies on the topic, none of which was conducted in Africa (27). One of them, a randomized controlled experiment conducted in 108 Canadian public health departments, identified research culture as an interaction variable, suggesting that KB would be more efficient in a context where the research culture is not strong (28).

Low- and middle-income countries have even more difficulty using research-based knowledge because of often limited access to scientific databases. This difficulty may be compounded in French-speaking countries, where few decision-makers are able to read English and where health needs are often greater. West African countries have more difficulty using research results, both because of limited access and because decision-makers there perceive the scientific content as presenting only partial conclusions, often in a format they find difficult to understand, and they generally do not possess the tools needed to consolidate the various results (29–32) . Thus, mechanisms are needed to put the knowledge into a format that is more appropriate to African decision-makers’ action priorities and ways of working (30). Research results uptake also requires a climate of confidence between researchers and users (33). However, decision-makers and researchers belong to generally disparate universes, and their lack of interaction is considered to be the main obstacle to knowledge use (15). Interactive knowledge transfer (KT) strategies, such as brokering, are considered to be the most effective way of overcoming this obstacle (31, 34–36) . They offer a collaborative approach to problem-solving that includes creating connections between knowledge producers and users to encourage the exchange of information (35). Brokers help bridge the gap between producers and potential users of research data.

For all these reasons, implementing a KB project appeared to us to be a promising means of encouraging research use in a West African context (27, 30). To our knowledge, this is one of the first interventions to test this approach in Africa.

In this article we describe the first year of implementation of a KB program that has been in operation in the district of Kaya, in Burkina Faso, since January 2012 and is planned to span 4 years (37). With knowledge brokers as intermediaries, this strategy builds upon personal contacts with decision-makers and health workers to integrate evidence-based knowledge more effectively into practice and decision making.

## Method

### Part 1: collaborative development of the KB strategy

The KB program was developed in several stages. First we conducted a scoping study (38), consulting the main databases,^2^ to ensure that the most recent evidence was taken into account in developing the KB strategy. This scoping review has been published in French in a public health journal (27). Using a grid developed iteratively, we analyzed 19 articles. The grid allowed collection of the following data for each article: type of documents, country of origin of the study, research objectives, type of brokerage activities, recipient of the intervention, type of study, and research methods. The synthesis showed that KB initiatives include: 1) planning activities (stakeholder identification, creation of networks and partnerships, context analysis, problem identification, and needs identification); 2) support to the brokers (training, technical support, and development of a practice guide); and 3) the brokerage activities themselves (information management, liaison between knowledge producers and users, and user training). This synthesis highlighted the challenges involved in KB activities, as well as the characteristics and skills that a broker should possess. Based on this work, we developed a policy brief about KB, in French,^3^ which we distributed to all partners in the research program (39).

**Fig. 1:**
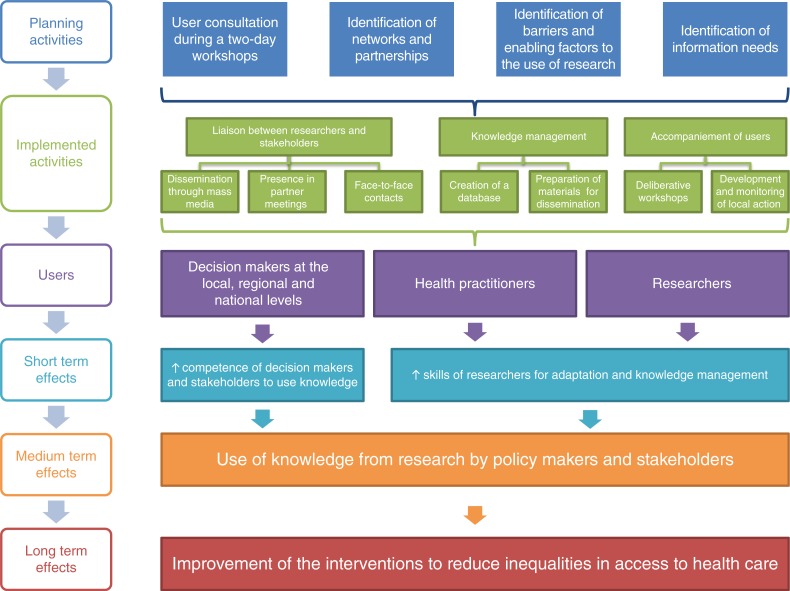
Logic model of brokering intervention.

Next, to validate the relevance of the proposed KB program in a Burkinabè context, we conducted two planning workshops in November 2011 with decision-makers and stakeholders (Ministry of Health, communes, NGOs, associations, etc.) from the national level (*n =*12) and the local and regional levels in the Kaya region and district (*n*=13). The aim of these workshops was to explore the issue of low research use and to adapt the KB strategy to the Burkinabè context. At these workshops, a draft version of the KB policy brief served as the basis for discussion to illustrate what we were planning, and we were able to fine-tune the plan based on what stakeholders considered relevant. The KB program was then represented graphically in the form of a logic model for the intervention (Fig. 1). This logic model shows that the process of KT between the broker and users (practitioners, decision-makers, and researchers) includes both planning and KB activities. Planning activities consist primarily of identifying key stakeholders, networks, and partnerships; analyzing the implementation context; and identifying the issues on which users would like to receive scientific information. The KB activities include liaison, information management, and support to users. The preferred liaison activities involve communications, both with individuals face-to-face, and in partner meetings. They also involve using local radio stations to disseminate messages to users at the community level. Information management activities involved developing documentary research strategies, setting up a database to facilitate access to relevant information, and drafting summary documents to respond to information needs (knowledge syntheses, policy briefs, and lay summaries). Lastly, activities to support users consisted of organizing deliberative workshops, participating in the development of action plans to change practices, and monitoring those action plans. The short-term objectives were to increase decision-makers’ and stakeholders’ competencies in using research and to develop researchers’ capacities to transform knowledge into a format suitable for transfer.

In November 2011 and January 2012, interviews were conducted to recruit a knowledge broker. Selection criteria included: 1) master's level training in either human and social sciences or public health; 2) at least 5 years of work experience in research or with researchers; 3) basic information technology competencies (Internet, word-processing and presentation software, etc.); 4) familiarity with reference management software (Endnotes, Mendeley, etc.); 5) excellent oral and written communication skills; and 6) ability to judge the quality of both quantitative and qualitative information. However, as there is no academic program to train knowledge brokers, we had to plan an intensive training program for this specific initiative. The knowledge broker works under the joint supervision of a senior consultant based in the capital and a researcher from the University of Montreal.

In January 2012, during a 2-day planning session held in Kaya, the practitioners, decision-makers, and research team met again. The results of that workshop served to orient the content of the research program and to identify the knowledge needs of the practitioners in order to guide the knowledge brokers’ work. Those 2 days also provided an opportunity to identify any needs for which there was already evidence that the knowledge broker could transfer while waiting for the researchers to produce results on the interventions in place in the region. For example, some participants wanted the impacts of user fees exemptions for healthcare to be evaluated, whereas several studies had already been carried out elsewhere in the country and in Africa on this subject. In this case, rather than evaluating the interventions, the knowledge broker prepared a 20-page narrative review of the literature.

Two Canadian experts trained several knowledge brokers in two 5-day sessions in January and May 2012 (Table 1). Both held administrative positions in healthcare organizations in Quebec. They had already developed and led training sessions on KB there and had several years of experience supervising teams of knowledge brokers. Given the investment required to train a broker and the 4-year time frame of the planned program, we decided to train several brokers as a hedge against the possibility that our knowledge broker might leave the project prematurely. Therefore, the training activities were also open to five professionals from certain partner research institutions in Burkina Faso. To date, we have not had to call on these professionals, as our knowledge broker is still in place. A 2-day introduction to KT was also offered to Burkinabè researchers from the team's institutional partners. In September 2012, a 2-week observation internship in Canada was organized to help brokers consolidate their new skills. This was an opportunity for the new brokers to spend time with KB specialists and share in their daily activities.

**Table 1:** Knowledge broker training program (10 days)

Block 1	
Day 1	General principles of knowledge brokering. Roles of a knowledge broker. Who uses knowledge brokers? Why use knowledge brokering? The expert and the knowledge broker: different but complementary. Conceptual model of knowledge management. Brokering products and processes.
Day 2	Documentary research. Information research: complementary resources. The steps of documentary research. The grey literature.
Day 3	The practice survey. Costs associated with a practice survey. Methods for identifying practices. Production of a practice survey report.
Day 4	Use of experts. Differences between consulting experts and surveying practices. Criteria for selecting experts.
Day 5	The broker's role in facilitating decision making. Components of a position paper. Qualities of a position paper. Steps involved in drafting a document to support decision making.
Block 2	
Day 1	Discussion on the importance of analyzing conditions. Overview of basic general conditions for facilitating new practices. Using strategies linked with the change curve. Analyzing specific conditions that can impede or facilitate implementation. Using the instrument for assessing conditions that can impede or facilitate implementation.
Day 2	Identifying the reasons for performing a needs assessment. Identifying objectives for data collection. Identifying the individuals targeted by data collection. Choosing the data collection method. Identifying what questions to ask. Mastering the art of asking questions. Analyzing and interpreting information.
Day 3	Writing in a succinct and accessible manner. Creating work aids (practice manual, summary, instrument, etc.).
Day 4	Specifying implementation objectives and the individuals targeted. Identifying strategies linked to the targeted objectives. Identifying strategies linked to the needs and conditions identified by the assessment instrument presented on day 1.
Day 5	Review of all material covered. Evaluating the impact of strategies implemented.

### Part 2: year 1 implementation evaluation of the KB program

The evaluation of the program's first year of implementation had three objectives: 1) to measure the reactions of knowledge brokers and researchers to the training activities; 2) to document KB activity implementation and output; and 3) to analyze the implementation processes.

Participants’ reactions to the training activities were evaluated using a questionnaire administered at the end of each day of training. This questionnaire was adapted from an instrument previously validated in a Quebec (French-Canadian) context (40). It captured participants’ reactions with respect to the training objectives and content, the organization of the sessions, the teaching approach used, and the trainers in general. Participants were also asked to identify which aspects of the training program they appreciated least or most, as well as which activities, in their opinion, required additional content.

KB activity implementation (Fig. 1) was measured by analyzing the broker's agenda and logbook. These tools were monitored weekly by both project supervisors to verify the quality of data entry and to ensure the brokering activities (development of policy briefs, PowerPoint presentations for workshops, research summaries, presentations to users, etc.) were being carried out.

The first author of this paper conducted semi-structured individual interviews (*n=*19) in French to gather information on factors that facilitated or impeded the implementation of KB activities. The broker and a representative sample of 18 potential users were chosen according to a purposeful sampling strategy (41). Notes were taken during the interviews, which were also audio-recorded. Each evening, the interviewer listened to that day's recordings to complete the notes. Then, another member of the evaluation team also listened to the recordings, adding to the notes as required to ensure the information was complete. The interview notes were subsequently analyzed using a methodology inspired by the first steps of grounded theory (42, 43). Following the principles of open coding, the main discursive themes in the interviews were identified and coded into concepts. The evaluation results were derived from descriptive analyses of the participant training session questionnaires and thematic analysis of the 20 qualitative interviews.

## Results and discussion

### Objective 1: reactions to training activities

Five individuals who were likely to use the knowledge and competencies imparted in the broker training attended the first week, and eight attended the second week. The results were very positive with respect to the sessions’ organization, content, and trainers (Table 2). All participants agreed that the workshop content fully met their expectations.

**Table 2:** Average level of participant satisfaction with various aspects of the three training sessions

	Knowledge broker training #1 (*n*=5)		Knowledge broker training #2 (*n*=8)		Researcher training (*n*=15)	
			
	Mean	s.d.	Mean	s.d.	Mean	s.d.
Objectives and content	3.8	0.23	3.6	0.3	3.6	0.44
Organization	3.8	0.06	3.7	0.01	3.7	0.11
Teaching approach	3.9	0.14	3.7	0.07	3.8	0.07
Trainers	4.0	0.02	3.9	0.03	3.9	0.06
Overall evaluation	4.0	0.11	3.9	0.10	3.9	0.26

Respondents particularly appreciated the practical exercises, the discussions, and the trainers’ interactive teaching approach. All participants agreed the information presented was quite new to them and would be useful in their work. However, some said there were not enough teaching tools. Respondents made very few suggestions for improvements; however, some would have liked more exercises on documentary research and evaluating scientific articles, whereas others would have preferred doing fewer exercises and focusing more on fully grasping the concepts.

Fifteen Burkinabè health researchers were invited to a 2-day training session on KT. They were very satisfied with the event. The average mean score for each dimension ranged from 3.6 to 4.0 on a four-point scale (Table 2). All participants agreed that the training met their expectations and that the information would be useful in their work. They agreed the trainer was well acquainted with the content, answered questions clearly, and made the workshop interesting. The participants were very satisfied with the section on developing policy briefs. However, many found the training session too brief and felt they did not have enough time to thoroughly understand the material and adequately perform the exercises.

These results show that the knowledge brokers and partner researchers considered the training activities to be relevant and useful. Although the training sessions were developed in Quebec, the fact that they were subsequently adapted to the realities of Burkina Faso with the people on the ground, undoubtedly explains the relevance of the proposed content. Given the paucity of indications in the literature on KB, we consider these results to be satisfactory and useful for others promoting KB projects.

### Objective 2: program implementation and outputs

The knowledge broker produced a substantial number of documents during the first year of intervention – in all, 11 reports. These included two literature reviews (specifically on obstacles to MHO membership and theoretical models of health behavior change) and reports on activities (Quebec internship, deliberative workshop, action plans, documentary research checklists, etc.). He also prepared more than 30 documents to disseminate the terms of reference of meetings, to list users’ high-priority questions, and to present various knowledge syntheses. Two deliberative workshops, both based on a literature synthesis and a policy brief, were organized to answer high-priority stakeholder questions (44, 45). The first, on MHO membership, had 15 participants. These were representatives from seven MHOs together with several local and regional decision-makers. The goals of this workshop were to share a summary of the determinants of low membership, inform participants about the broker's role, and identify coaching techniques that could be used in developing and implementing action plans. This workshop prepared the way for three other meetings to develop action plans and follow up on them. The second workshop took place in January 2013 and focused on theoretical models for understanding health behavior – a crucial subject in a context where prevention projects are often difficult to implement. The goals of this workshop were to share an overview of health behavior with participants and local decision-makers, inform them of the broker's role, and raise their awareness of the importance of supporting decision making with evidence-based data. Fourteen practitioners and decision-makers from several organizations involved in healthcare (NGOs, health district, regional health department, mayor's office, etc.) attended this workshop.

Monitoring charts showed that some 50 different individuals participated in at least one KB activity (deliberative workshops, priority issue meetings, etc.). These individuals came from research institutes, various Ministry of Health agencies and local and regional authorities (health district, regional health departments, etc.), local and international NGOs, and MHOs.

**Fig. 2:**
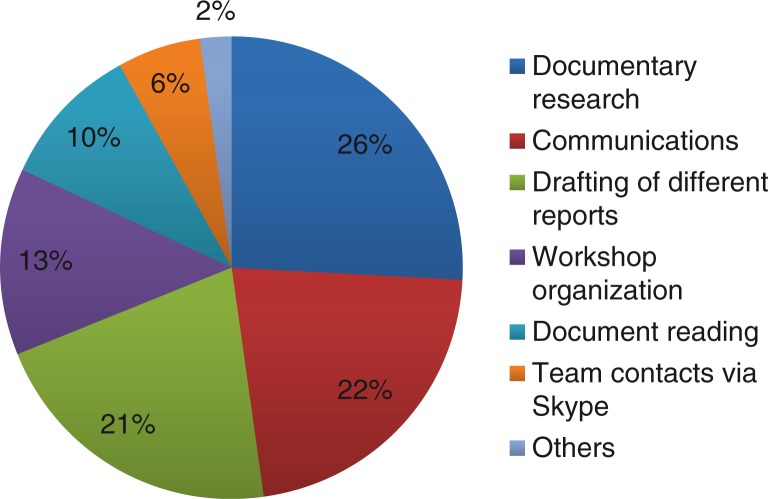
Proportion of time invested by the junior broker in each activity.

Figure 2 summarizes the broker's use of time between May 2012 and January 2013. Documentary research occupied the greatest proportion of his time, because he experienced difficulty in applying the techniques for developing a documentary research strategy taught during the training. He required some long-distance coaching by the knowledge management expert, with whom he developed and tested many queries before validating them. Email correspondence took 22% of the broker's time. Communications with different stakeholders, including supervisors, occurred all day, every day, and were necessary to ensure information circulation to both users and partners. One-fifth of the broker's time was spent drafting reports and documents. Organizing workshops involved a certain amount of advance work, that is, sending out invitations, preparing budgets, booking venues, organizing breaks, preparing PowerPoint presentations, printing and copying documents, preparing mail shipments, following up, and confirming with participants. The remainder of the broker's time was spent reading documents and contacting team members via Skype.

There are very few references available on the implementation of KB strategies. This explains why, when the present program was implemented, no targets were set in terms of quantity of documents to be produced or contacts with stakeholders. Even though it took 5 months of preparation before the broker offered the first KB activity to users, we consider the results of this component of the evaluation to be satisfactory. When organizing an innovation in such a context, it is important to take the time to understand the issues well and to explain the details of the action to all the stakeholders. This was essential in our case, as not all the parties involved were familiar with the concept of KB. The results of this intervention could also serve as a point of reference for other KB projects in the future.

### Objective 3: analysis of implementation processes

The implementation analysis results were based on discussions with respondents around three main themes: 1) the usefulness of the knowledge transferred by the broker; 2) their assessment of the intervention's strengths and weaknesses; and 3) their opinions about how the program might be improved in subsequent years. The results presented in this section are also based on our observations of the broker's implementation, training, and coaching activities.

#### Usefulness of the knowledge transferred

All respondents had attended at least one formal broker-organized activity. Their opinions of the program were unanimously positive. While the decision-makers considered that research was useful and should be used in both practice and decision making, the respondents actually citing the most examples of the usefulness of knowledge transferred by the broker were from NGOs and MHOs: ‘Research has contributed to MHO development; [for example, it helps in understanding] why people don't join, what is this related to? It helps in understanding why and in finding solutions’ (MHO respondent). Likewise, ‘this question [on models of health behaviour change] was formulated by NGOs but could also be used by government agencies, because awareness-raising activities are often carried out with no specific model, on the fly: models are needed to do things better’ (NGO respondent).

#### Program strengths and weaknesses

To some MHO respondents and most of the decision-makers encountered, the program's objectives and the broker's role were still sometimes muddled. Some wanted the KB program to generate supplementary financial resources: ‘… if the program has the funds, take charge of paying their premiums [to the MHO]’ (MHO respondent). Others, however, understood clearly that this was not the intended role: ‘… they need to understand that this is what's being provided; people need to understand why there aren't any per diems, but that, in return, the broker works for them and gives them relevant new information’ (NGO respondent).

The majority of MHO respondents were very enthusiastic about participating in these activities and saw the broker-led activities as being very useful. They offered many examples: ‘He gave us ways of increasing membership, he prompted us to develop an action plan, and he coached us through its implementation’ (MHO respondent). For most of them, participating in these activities led to concrete actions in the field, such as awareness-raising outings and mobilization of influential people in the community, that generated increased membership.

Respondents noted a few of the program's limitations. Mainly, they thought there should be more meetings and more regular contacts with the broker in the field: ‘… ongoing contact, even if there isn't an upcoming workshop; we shouldn't wait for workshops to speak to people …’ (NGO respondent). A few participants in a workshop on health behavior models found this material more difficult to understand and suggested that the language level should sometimes be adjusted to avoid overly technical terminology.

Respondents identified several strengths in the KB program. Several mentioned that the questions addressed during the activities met a need expressed by stakeholders. The broker's and speakers’ competencies were also affirmed: ‘Speakers and facilitators mastered their fields. In fact, they were able to lead the workshop without reading their papers, and to add comments and adjust [to the context]’ (decision-maker respondent). A few respondents noted the broker's versatility, which enabled him to obtain the information they needed. Lastly, another major strength was the fact that the participation level was high and sustained, even without per diems, which is an issue in Burkina Faso (46).

#### How the program might be improved

Finally, several suggestions were made for improvements in subsequent years. One respondent pointed out the need to connect with decision-makers at the central level: ‘This has to go up to the central level, which hasn't yet happened, but there used to be relatively few products to disseminate. Now that there is more to present, we have to go there’ (local decision-maker). Another challenge related to the issue of inadequate resources had to do with coaching from the broker; while participants recognized he was not there to provide financial support directly, they nevertheless hoped that ‘… the broker could support us in our search for funding’ (MHO respondent). Lastly, some mentioned the need to ensure the program's sustainability, adjust the level of language, and increase the broker's presence in the field.

Despite the increasingly widespread use of brokers to create a bridge between the research and practice settings to promote the use of scientific knowledge, this role remains poorly defined (47–50) . Brokering can take many forms and can be done in person, by telephone, or over the Internet. The frequency of interactions can range from daily, in some cases (51), to as little as once every 2–3 months, in others (22, 52). Furthermore, despite recent efforts to document the broker's role (50, 53–55) , there is still very little evidence on the activities that brokers should perform in the different contexts to which they are assigned. As noted by Conkling and colleagues (56), ‘… the role is difficult to define, emergent, abstract, episodic, and not fully understood’ (p. 1). A few articles published over the past 5 years have provided information on the distribution of the broker's tasks (56, 57) and on the implementation of this strategy (58), but none have described the process of planning a brokering program, the training activities that need to be organized, and the different stakeholders’ roles in its development. We believe it is in these areas that our article offers a significant contribution.

## Conclusion

The first section of this article described the different stages in the development of an intervention considered to be increasingly relevant in closing the gap between research, practices, and decision making. The implementation evaluation results showed that the preparatory activities, and in particular the training provided to knowledge brokers and researchers, were appreciated by participants, and that most of them considered that the content covered would be helpful in their work. Still, many would have liked more hours of training. A review of program activities and outputs also showed that, by the end of the first year, the broker had assumed his role in accordance with the logic model. Analysis of the interviews showed that this role was deemed important by the participants targeted by those activities and outputs.

In this context where research use culture is still largely undeveloped, the coaching provided by the broker appears to us to be virtually indispensable. The broker's role cannot be limited only to transforming and disseminating information: it needs to include coaching that is responsive to change. We cannot yet, from the results of the evaluation of this first year of activity, draw any conclusions about the approach's efficacy in changing practitioner and decision-maker behavior. Nonetheless, this first stage in the process evaluation is promising: the intervention was appreciated and adapted to the context, and the results remind us of the extent to which evidence is poorly known and inaccessible. This, in turn, reinforces the relevance of the KB program.

The program in Burkina Faso is a complex system made up of several activities aimed at a variety of interdependent actors (researchers, users, brokers) whose views about research are sometimes divergent. The West African context is another factor that adds to the complexity and must not be neglected when attempting to understand the program's functioning and effects. As such, a great deal of attention should be given to the context, the complexity, the nuances, and the interdependence of factors motivating behaviors. For example, in a country where decision making is highly centralized, any influence exerted to effect change should not be limited to the local arena, but should definitely also focus on the national level. For our project, this will involve determining how to disseminate knowledge more effectively to influence change at the national level. We believe it is important, before implementing any KB intervention, to analyze the decision-making context carefully. Likewise, in a country that receives a great deal of international aid for development, staff turnover is a major problem, as professionals change programs often and officials in various ministries have often been corrupted by NGOs and international aid agencies. It is therefore important to train as many brokers as possible and to maintain close ties with decision-makers.

A comprehensive evaluation of the program is currently under way. Given the program's complexity, the evaluation is targeting several aspects. Our aim is not only to continue describing the activities undertaken and their impacts on the actors, but also to explain the effects obtained, while also studying the potential unintended effects. Thus, every component of the logic model will be evaluated to arrive at a complete, dynamic, and comprehensive understanding of the program.

In the long run, the evaluation of the brokering program will provide knowledge on the broker's roles and responsibilities and on this strategy's effects on the use of research results to improve population health.

## Ethical considerations

Ethics certificates were obtained from the research ethics committees of the University of Montreal Hospital Research Centre (12.273) and the National Health Ethics Committee of Burkina Faso (2012-11-85).

## Footnotes

1 MHOs are community-based organizations set up by social groups to facilitate their members’ access to good quality healthcare services.

2 ERIC, MEDLINE, psycINFO, santécom, BDSP, CINAHL, and Business Source Premier.

3 Because French is the primary language used in Burkina Faso, all KB and evaluation activities were conducted in French.
